# Regulation and dysregulation of hair regeneration: aiming for clinical application

**DOI:** 10.1186/s13619-022-00122-x

**Published:** 2022-07-01

**Authors:** Zhicao Yue, Fang Yang, Jianglin Zhang, Ji Li, Cheng-Ming Chuong

**Affiliations:** 1 Department of Cell Biology and Medical Genetics, International Cancer Center, and Guangdong Key Laboratory for Genome Instability and Disease Prevention, Shenzhen University, A7-455 XiLi Campus, Shenzhen, 518060 Guangdong China; 2 Department of Dermatology, Shenzhen People’s Hospital, The Second Clinical Medical College, Jinan University, The First Affiliated Hospital, Southern University of Science and Technology, Shenzhen, Guangdong China; 3 Department of Dermatology, Xiangya Hospital, Central South University, Changsha, China; 4 Department of Pathology, University of Southern California, Los Angeles, CA USA

**Keywords:** Hair follicle, Stem cells, Regeneration, Androgenetic alopecia, Alopecia areata

## Abstract

Hair growth and regeneration represents a remarkable example of stem cell function. Recent progress emphasizes the micro- and macro- environment that controls the regeneration process. There is a shift from a stem cell-centered view toward the various layers of regulatory mechanisms that control hair regeneration, which include local growth factors, immune and neuroendocrine signals, and dietary and environmental factors. This is better suited for clinical application in multiple forms of hair disorders: in male pattern hair loss, the stem cells are largely preserved, but androgen signaling diminishes hair growth; in alopecia areata, an immune attack is targeted toward the growing hair follicle without abrogating its regeneration capability. Genome-wide association studies further revealed the genetic bases of these disorders, although the precise pathological mechanisms of the identified loci remain largely unknown. By analyzing the dysregulation of hair regeneration under pathological conditions, we can better address the complex interactions among stem cells, the differentiated progeny, and mesenchymal components, and highlight the critical role of macroenvironment adjustment that is essential for hair growth and regeneration. The poly-genetic origin of these disorders makes the study of hair regeneration an interesting and challenging field.

## Background

The cyclic growth and regeneration of hair follicles (HFs) is a prominent example of organ regeneration in our body. From over 1 hundred years ago, radiation-induced HF damage and regeneration (Williams 1906; Coolidge 1925; Chase 1946; Potten 1970; Malkinson and Keane 1981; Inomata et al. 2009; Huang et al. 2017), and later on chemotherapy-induced hair loss (Malkinson et al. 1961; Paus et al. 1994; Haslam et al. 2021), have led to the concept of epithelial and melanocyte stem cells. Early surgical experiments on rat vibrissae follicles examined the contribution of epithelial and mesenchymal components that are essential for HF regeneration, and established the HF as a paradigm of tissue interactions in organ regeneration (Oliver 1966, 1967). In the 1990s, the HF stem cells (HFSCs) were localized in the lower part of the permanent portion of the follicle, the so-called bulge region, based on their slow-cycling thus label retaining property (Cotsarelis et al. 1990), and clonal growth capability (Rochat et al. 1994). With the availability of more powerful genetic tools, the characteristics of these stem cells have been further explored (Gonzales and Fuchs 2017; Rognoni and Watt 2018). Particularly in recent years, the concept of macro-environment regulation of HFSC has attracted much attention (Chen et al. 2016; Plikus et al. 2008, 2011; Chen et al. 2015). These include the surrounding adipose tissue (Plikus et al. 2008; Festa et al. 2011; Zhang et al. 2016), immune systems (Chen et al. 2015; Ali et al. 2017), vascular system (Li et al. 2019; Gur-Cohen et al. 2019; Peña-Jimenez et al. 2019), mechanical force (Xie et al. 2022; Koester et al. 2021), neuroendocrine factors (Choi et al. 2021), and environmental factors such as temperature (Shwartz et al. 2020) and dietary factors (Morinaga et al. 2021). Thus the molecular regulation of hair growth and regeneration has seen rapid progress in recent years.

One reason why hair growth and regeneration has attracted much attention is because hair loss remains an outstanding health problem. Although not lethal, the disfiguring and psychological pressure makes hair loss a major concern for adult male and female as well. Rare genetic mutations have been associated with hair disorders (Ahmed et al. 2019), however, for the majority of hair disorders such as male patterned hair loss (androgenetic alopecia, AGA) and alopecia areata (AA), the genetic factors have not been well defined (Heilmann-Heimbach et al. 2016; Pratt et al. 2017). Recently, genome-wide association studies (GWAS) described the susceptible loci for AGA (Hagenaars et al. 2017; Pirastu et al. 2017; Heilmann-Heimbach et al. 2017; Yap et al. 2018; Li et al. 2012a), and multiple immune-related genes have been associated with AA (Petukhova et al. 2010, 2020; Betz et al. 2015). It is clear that both AGA and AA are complex diseases with multiple genetic risk factors, yet how these genetic factors are related to disease initiation and progression remains largely unknown. Further research is required to define the pathobiology of these complex diseases, and develop novel therapeutic strategies accordingly.

Here, we summarize recent progresses in our understanding of hair regeneration, with an emphasis on the complex regulation of HFSCs. We wish to highlight that hair regeneration represents an attractive model for the mechanistic study of organ regeneration and the functional involvement of stem cells. Moreover, the knowledge gap between the GWAS results and the pathological mechanisms of AGA and AA poses a major challenge in the field.

## The improved bulge activation hypothesis

**Fig. 1:**
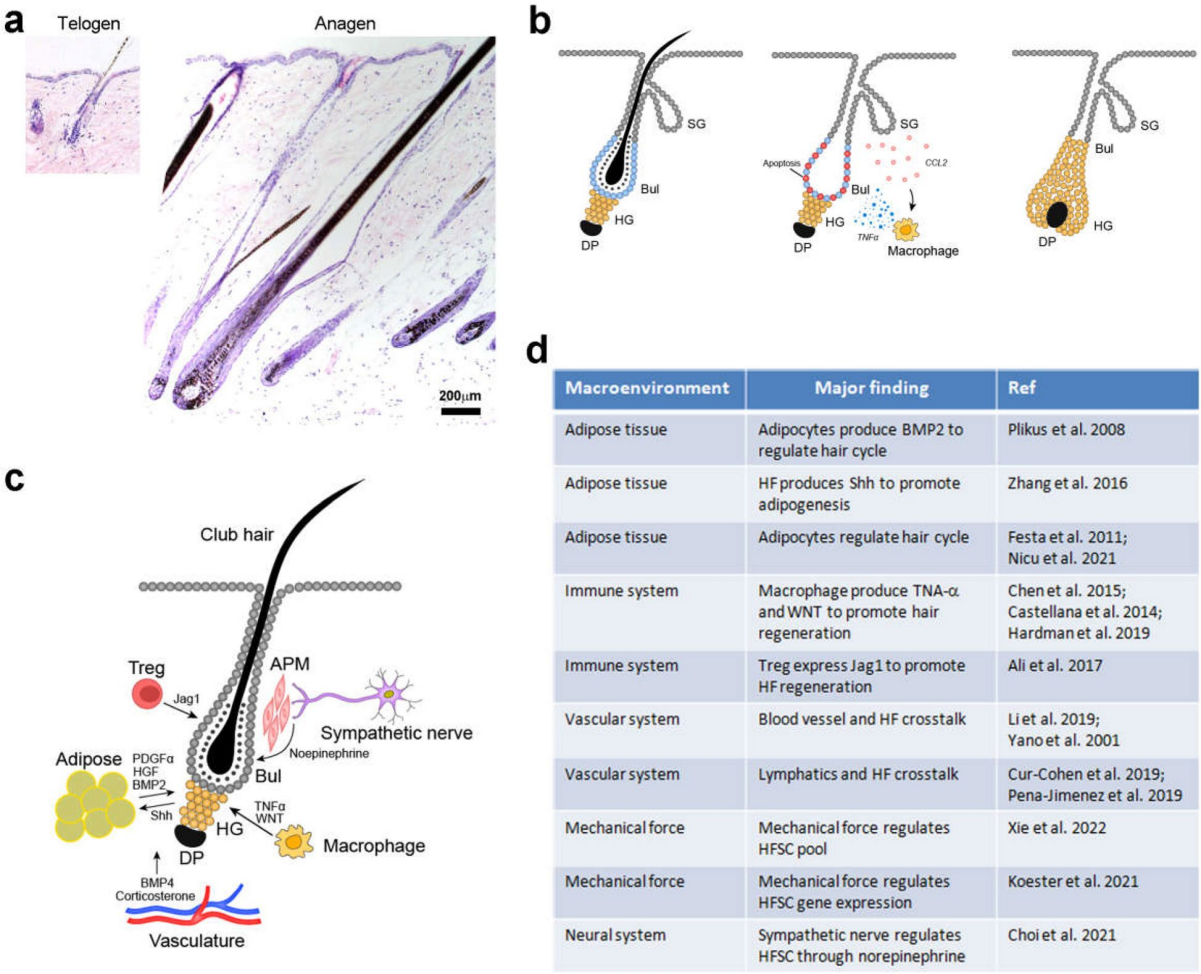
Regulation of hair regeneration. **A** Haematoxylin and eosin staining showing the mouse HF in resting (telogen; left) and growing (anagen; right) phases. The panels are of the same magnification to show the difference in size. **B** Diagrams showing the hair regeneration process. The hair germ (HG) is the population of progenitors between the dermal papilla (DP) and the bulge stem cells (Bul). Plucking induces bulge stem cell apoptosis, and Ccl2 secretion from the wound epithelium, followed by activation of macrophage which secrets TNFα to promote hair regeneration. SG, sebaceous gland. **C** A diagram summary of the various layers of regulation on hair regeneration. Sympathetic nerves produce norepinephrine to activate HFSCs in the bulge (Bul). Macrophages produce TNFα (in wound) and Wnt ligands (in natural telogen-anagen transition) to activate hair growth. Adipocyte precursors secret PDGFα/HGF to promote hair growth, and adipocytes produce BMP2 to help maintain telogen, whereas these cells sense SHH produced by actively growing HFs to proliferate. Treg cells express Jag1 to activate Notch signaling and HFSCs. The vasculature systems including blood vessels and lymphoid vessels are also important for hair growth and regeneration. Circulating hormones such as corticosterone act on the DP to regulate hair growth. **D** Summary of the various macro-environmental factors regulating HFSCs and hair regeneration

As an easily accessible model, the mouse hair is perhaps the most studied example of hair growth and regeneration. Its growth cycle in adult life can be divided into actively growing (anagen), regression (catagen), and resting (telogen) phases (Fig. 1A; Hardy 1992). One can immediately appreciate the differences in these stages: the length and size of the HF undergo dramatic remodeling during its life cycle. The mouse hair is usually of limited length because the duration of anagen is under stringent control, and most of the time it is in telogen (Hardy 1992). The molecular clock(s) for the transition of these different growth phases remains largely unknown. A natural mutation on the *Fgf5* gene leads to longer hair in the ‘angora’ mouse strain (Hébert et al. 1994; Harshuk-Shabso et al. 2020). Human HFs also undergo cyclic growth and have anagen, catagen and telogen, just as the mouse counterparts (Paus and Cotsarelis 1999). However, the human HFs can have a much longer anagen which then result in much longer hair, the longest being over 5 m according to the Guinness World Records [https://www.guinnessworldrecords.com/world-records/longest-hair-(female)].

The epithelial stem cells of the HF reside in the bulge region which is the lower part of the permanent portion (Cotsarelis et al. 1990; Rochat et al. 1994). This has led to the so-called “bulge activation hypothesis” that is proposed to account for the hair growth cycle: in each cycle, the stem cells produce transient amplifying cells that actively proliferate to build the hair, the exhaustion of which then leads to HF regression and catagen/telogen. This theory appears elegant and is indeed substantiated by multiple tracing methods during natural hair cycle (Taylor et al. 2000; Tumbar et al. 2004). However, several lines of evidence suggests that there are still missing links.

1. The molecular determinants for the duration of anagen remain unclear. The concept that “exhaustion” of the transient amplifying cells is a vague term and we know little about how this process is controlled at the molecular level, which is central for hair grow control.
2. In plucking-induced hair regeneration, the majority of epithelial HFSCs undergo apoptosis, a phenomenon confirmed by several studies (Ito et al. 2002, 2004; Chen et al. 2015). An explanation is that the lower bulge collapses and re-populates the hair germ (HG) and stem cell population during the regeneration process (Ito et al. 2004). Thus the HFSCs are under dynamic regulation and not a fixed population.
3. Even for this simple plucking-regeneration scenario, it has been shown that hair regeneration is not controlled at the single follicle level; rather, a collective behavior has been documented which states that regeneration is controlled at the population level and a quorum sensing mechanism is involved (Chen et al. 2015). Indeed, plucking an individual HF in mouse skin will not lead to regeneration; rather, regeneration is triggered only after the damage signal accumulates to overcome a threshold by plucking a certain amount of HFs. This process is coordinated by the crosstalk between HFSCs and the skin immune system (Fig. 1B; Chen et al. 2015).
4. The HG is an intriguing twist on the HFSC concept. Although it has long been known that a specific population of progenitors are critical for hair regeneration, the molecular nature of the HG is only recently been examined by profiling, single cell RNA-sequencing and lineage tracing (Greco et al. 2009; Rompolas et al. 2012, 2013; Yang et al. 2017; reviewed in Panteleyev 2018). These cells are not stem cells but are likely to be responsive for communicating with neighboring cells and the formation of the cycling HF (please see Panteleyev 2018 for more discussion).
5. Classical experiments have established the critical role of the dermal papilla (DP) in hair growth and regeneration; however, it has also been documented that the DP together with the lower one-third of the follicle can be regenerated (Oliver 1966, 1967; Rahmani et al. 2014). In addition, the HFSCs can be regenerated from residue cells during regeneration (Ito et al. 2004; Rompolas et al. 2013). Thus the HFSCs represent a functional status and can be induced during regeneration, just like the stem cells in the intestinal villi (Guiu et al. 2019; Murata et al. 2020; Liu et al. 2020).

Based on the current data, it appears that the bulge activation hypothesis which accurately placed the role of HFSCs, is complicated by the multiple layers of regulation on the various aspects of hair growth and regeneration. It is intriguing to consider the possible differences between natural hair cycle and pluck-induced regeneration, and the fate of the bulge stem cells and the HG. Also, a global level of HFSC regulation both in terms of the HF population, and the multiple cell types within the skin is of major interest.

## Toward the macroenvironment that regulates hair regeneration

Recently, interesting progress has been made on the complex regulation of hair growth and regeneration, with an emphasize on the crosstalk among the multiple cell types in skin and environmental factors (Fig. 1C and D). For instance, cold temperature was shown to impact HFSCs through a neuroendocrine mechanism, specifically norepinephrine from the sympathetic nerves promotes HFSC activation thus hair regeneration (Shwartz et al. 2020). Another layer of neuroendocrine regulation regards the corticosterone from the adrenal gland which acts on the DP and inhibits the secretion of Gas6 and reduces the activity of HFSCs (Choi et al. 2021). Interestingly, norepinephrine from the sympathetic nerves but not the adrenal gland depletes hair follicle melanocytes and leads to hair greying (Zhang et al. 2020).

Dietary fat reduces the activity of HFSCs through an oxidative mechanism and impacts the hedgehog signaling pathway, which may link obesity with alopecia (Morinaga et al. 2021). The contribution of dermal adipose tissue to HF formation and regeneration has also been concerned. Mature adipocytes express BMP2 and inhibit hair regeneration (Plikus et al. 2008), whereas adipocyte precursor cells produce PDGFα and promote HFSC activation and hair regeneration (Festa et al. 2011; Schmidt and Horsley 2012; Driskell et al. 2014). Adipocytes also secret HGF to promote hair growth and pigmentation (Nicu et al. 2021). Conversely, growing HFs produce SHH which acts on adipocyte precursors to promote dermal adipogenesis (Zhang et al. 2016). Interestingly, the Africa spiny mice (*Acomys*) which have extraordinary skin regeneration capacity including HF neogenesis (Seifert et al. 2012), have a very high contend of skin adipose tissue (about 85% full thickness of skin; Jiang et al. 2019). It is intriguing to consider the possibility that the skin adipose tissue contributes to the extraordinary regeneration capability of Spiny mice.

The critical contribution of the skin immune system in HF regeneration is nicely illustrated by the involvement of macrophage and TNFα secreted by these cells: the crosstalk began when the HF was plucked, which resulted in Ccl2 production from the epithelia as a wound signal (Chen et al. 2015). Skin macrophages also produce Wnt7b and Wnt10a and programmed for apoptosis during telogen-to-anagen transition, which is critical for HFSC activation and hair regeneration (Castellana et al. 2014; Hardman et al. 2019). In addition, regulatory T cells promote HF regeneration through the production of the Notch ligand Jag1 (Ali et al. 2017; Moreau et al. 2021). For more discussion on the immune aspects of HF regeneration please refer to Rahmani et al. 2020 and Naik et al. 2018.

The vasculature system is a critical component of the stem cell niche in many models, and the HFSC is no exception. In addition to supplying nutrition, the blood vessels also regulate the size of the HF (Mecklenburg et al. 2000; Yano et al. 2001). A specialized vascular annulus is permanently associated with the HFSC compartment regardless of its cyclic growth and regression status (Xiao et al. 2013). Paradoxically, a transient vascular plexus was identified during catagen/telogen phase of the hair cycle, which blocks HFSC activation via secreting BMP4 (Li et al. 2019). In addition, the lymphatic vessels are also involved in HFSC regulation which expand and shrink coordinately with the hair growth cycle (Gur-Cohen et al. 2019; Peña-Jimenez et al. 2019).

Together, it is clear that the regulation of hair growth and regeneration is much more complex than expected, which includes neuroendocrine factors and environmental factors such as temperature and dietary factors, and involves crosstalk among the several cell types within the skin including the adipose tissue, immune cells, and the vascular system (Fig. 1C). These results also point to the fact that the HFs represent a sensitive model to explore principles of organ regeneration and the involvement of stem cells.

## Dysregulation of hair regeneration in AGA

**Fig. 2:**
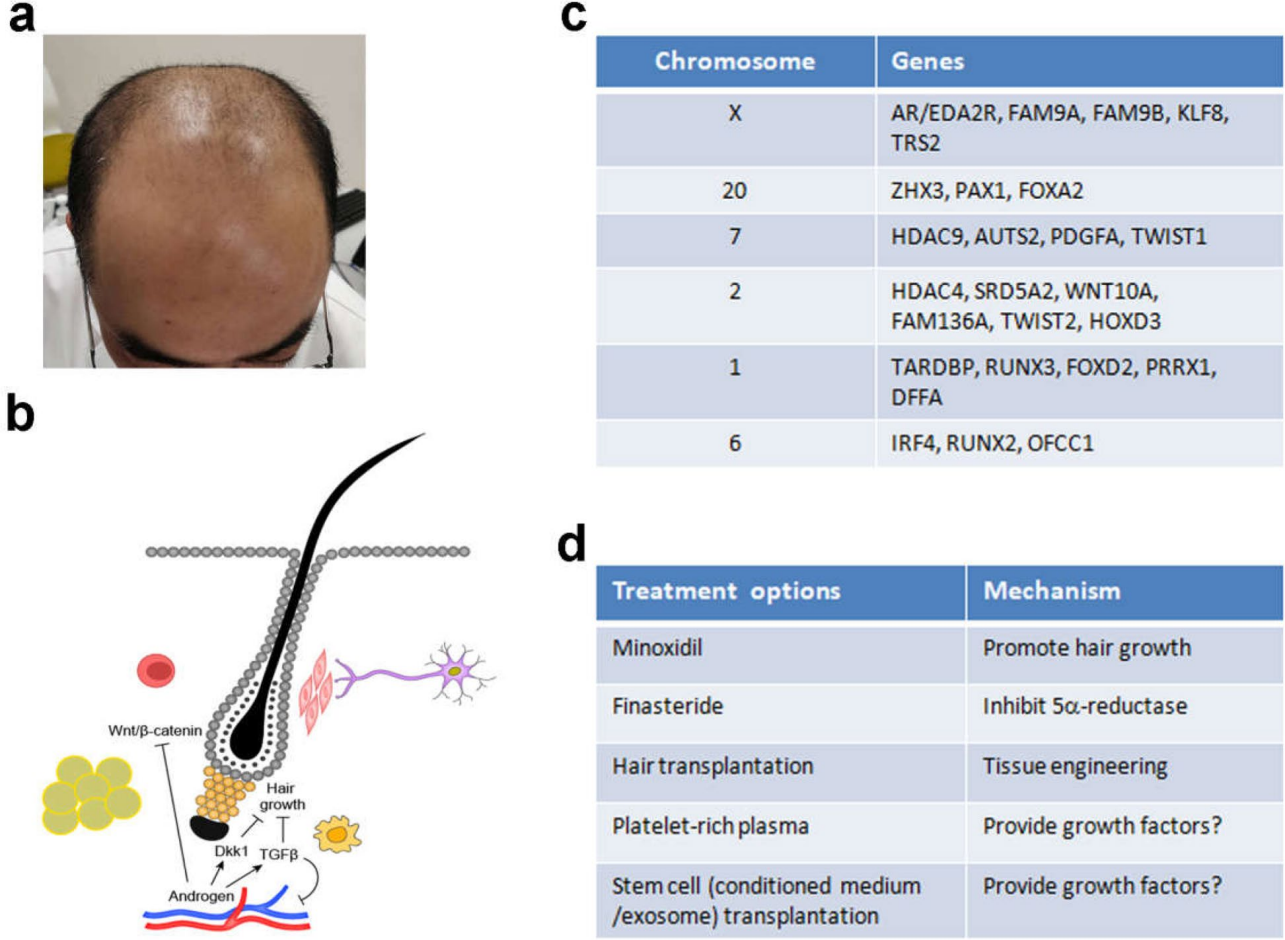
Dysregulation of hair regeneration in AGA. **A** Clinical presentation of a patient with AGA. **B** Androgen receptors (AR) in the hair follicle dermal papilla sense androgen and promote Dkk1 and TGF-β expression, which retard hair growth. TGF-β further shrinks the blood vessel. AR signaling also antagonizes Wnt/β-catenin signaling in the HF. **C** GWAS results showing the potential genes involved in AGA and their chromosomal locations. **D** List of current treatment options for AGA and the possible mechanisms

AGA, or male pattern baldness affects 30–50% men at the age of 50, and 80% men at the age of 80 (Fig. 2A; Hamilton 1951; Wang et al. 2010; Ding et al. 2020). Thus it is a common trait in the population. From the types and occurrence of AGA, there is a clear contribution of patterning, that is, HFs in the front/top region of the scalp are mostly perturbed. The molecular nature of this regional effect remains obscure. There is also the contribution of ageing, which may or may not add up to the key role of hormonal factors. In recent years, rapid progress has been achieved to define the genetic factors contributing to AGA (Hagenaars et al. 2017; Pirastu et al. 2017; Heilmann-Heimbach et al. 2017; Yap et al. 2018; Li et al. 2012a). These findings will likely impact the AGA pathobiology research in the future.

The pathological change of AGA is closely associated with hair cycle, namely the anagen-catagen-telogen transition. The hair becomes smaller and smaller as it cycles, the so-called terminal to vellus hair transition. Reflecting on this, there is the hypothesis that the DP cells may die, escape, or fail to replenish from the connecting dermal sheath, thus the diminished HF size (Jahoda 1998). Since the miniaturization of the HF seems to occur mainly on the telogen to anagen transition, that is, the onset of each hair growth cycle, the critical contribution of the epithelial components and the epithelial-mesenchymal interactions has yet to be thoroughly investigated. It was found that the KRT15+ epithelial stem cells are largely preserved in AGA, whereas the more active progenitors which are CD200hi ITGA6hi and CD34hi are greatly diminished (Garza et al. 2011). Therefore, it appears AGA is not a disorder of stem cells, but rather a dysregulation of stem cell to progenitor cell transition. Consistently, K14-Cre mediated *Dnmt1* knockout in skin leads to progressive hair loss whereas the K15+ stem cells are unperturbed (Li et al. 2012b; Chen and Chuong 2012). This study not only provides a model for progressive alopecia, but also conceptually supports the aforementioned AGA mechanism.

The key role of androgen and its converting enzyme 5-α-reductase which converts testosterone to the more active dihydrotestosterone has been well-documented (Lolli et al. 2017; Randall 2008; Inui and Itami 2013). The opposing role of androgen in the beard and scalp HF has also attracted some attention. It is believed that the local microenvironment including the receptors, hormone metabolism, and HF properties control the different responses: while the beard HFs are stimulated to grow and enlarge, the scalp HFs diminish in size (Lolli et al. 2017; Randall 2008; Inui and Itami 2013). The inhibitory factors downstream of hormone and 5-α-reductase include *TGF-β1*, *TGF-β2*, *IL-6* and *Dkk1* (Inui and Itami 2013), whereas *WNT5A*, *WNT10B* and *BMP2* are suppressed (Leirós et al. 2017; Ceruti et al. 2021) (Fig. 2B). On the other hand, androgen induces IGF-1 expression which then stimulates beard HF growth (Inui and Itami 2013). Thus any model that accounts for the impact of hormone should reconcile these different responses. For instance, androgen receptor signaling antagonizes Wnt/β-catenin signaling in mouse skin yet the regional factors remain unclear (Kretzschmar et al. 2015). Prostaglandin D2 may also contribute to follicular miniaturization and AGA (Garza et al. 2012). Another recent work documented that androgen receptor signaling in the HF induces TGF-β expression which then inhibits angiogenesis (Deng et al. 2022). Thus the link between the vascular system and HF regeneration was highlighted in AGA.

The availability of large-scale genetic databases allows the rapid progress of AGA genetics. GWAS analysis based on the UK biobank data identified 250 independent loci for severe hair loss, which correspond to 112 autosomal genes and 13 X-chromosome genes (Hagenaars et al. 2017). Another report claimed 71 loci of which 30 were novel can explain 38% of the AGA risk (Pirastu et al. 2017). Yet another report claimed 63 loci including 23 new ones can explain 39% of the phenotypic variance in AGA (Heilmann-Heimbach et al. 2017). The candidate genes include *FGF5*, *IRF4* and *DKK2*. In a later report, 624 near-independent loci were discovered, of which 26 X-chromosome loci explains 11.6% (Yap et al. 2018). Among the most often identified genes, androgen receptor (*AR*) which localizes on the X-chromosome showed the most significant association. Many of these loci are associated with the WNT signaling (*LGR4, RSPO2, WNT3, WNT10A, SOX13, DKK2, TWIST1, TWIST2, IQGAP1, PRKD1*) and apoptosis pathway (*BCL2, DFFA, TOP1, IRF4, MAPT*), with a third group linked to androgen and TGF-β signaling (*RUNX1, RUNX2, RUNX3, PTHLH, ALPL, AR, PDGFA, SRD5A2, FGF5, PAX3*) (Pirastu et al. 2017; Heilmann-Heimbach et al. 2017). Some of these genes are listed in Fig. 2C. Thus AGA is clearly a genetically based trait of multiple origins.

Unfortunately, the current treatment options for AGA are very limited (Fig. 2D). Minoxidil, which promotes hair growth, is widely used in various brand names yet the exact mechanism of action remains unclear. Finasteride, an inhibitor of 5-α-reductase, is often used in an oral format. Alternatively, hair transplantation is a viable choice. Other options include platelet-rich plasma (Shapiro et al. 2020; Qu et al. 2021) and transplantation of multi-potent stem cells of various origin or conditioned medium/exosome (Egger et al. 2020; Yuan et al. 2020), possibly by providing additional growth factors. None of these options are ideal which urges further research and development in this field.

## Dysregulation of hair regeneration in AA

**Fig. 3:**
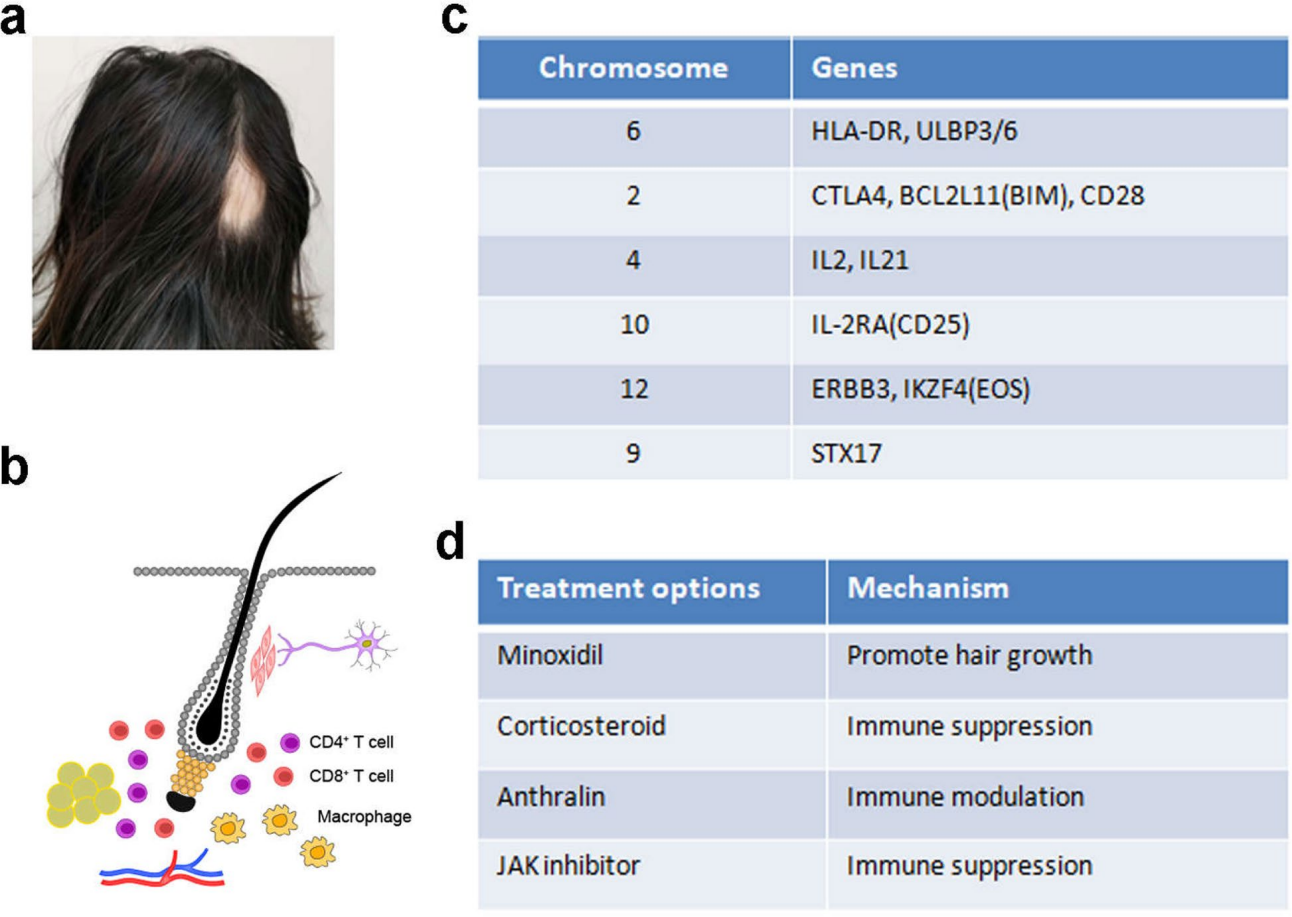
Dysregulation of hair regeneration in AA. **A** Clinical presentation of a patient with AA. **B** The pathobiology of AA involves immune cells attacking the lower part of the growing HF, which include expanded population of antigen-presenting macrophages, CD4+ T cells and CD8+ T cells around the HF. **C** GWAS results showing the potential genes involved in AA and their chromosomal locations. **D** List of current treatment options for AA and the possible mechanisms

AA is another common hair disorder that impacts 2% of the population (Pratt et al. 2017; Gilhar et al. 2012) (Fig. 3A). AA is often characterized as patched hair loss, although whole-scalp hair loss (AA totalis) or even whole-body hair loss (AA universalis) is possible. This type of hair loss is often reversible, and is caused by the immune system attacking the lower part of the HF (Fig. 3B; Pratt et al. 2017; Gilhar et al. 2012). Rapid progress has also been achieved in dissecting the genetic and pathological mechanism of the disease (Petukhova et al. 2010, 2020; Betz et al. 2015), although it is well appreciated that environmental factors and physical/psychological factors contribute significantly to trigger AA (Pratt et al. 2017). Novel treatment options are currently under extensive clinical test (Pratt et al. 2017; Gilhar et al. 2019; Paus 2020).

A key concept in AA regards the the immune privilege of the HF (Bertolini et al. 2020; Paus et al. 2018): the HF epithelial cells express lower level of MHC I and MHC II molecules which reduce their immonogenecity; there is increased expression of immune-inhibitory molecules such as CD200 and PD-L1; the immune-suppressive local factors such as αMSH, TGFβ1, TGFβ2, IL-10, MIF are also protective; other local factors may include neuropeptides and neurohormones, CD4+ regulatory T cells, and peri-follicular mast cells. When this immune privilege collapses, the lower HF is subject to attack by CD8+ T cells or NK cells. Inflammatory factors such as IFNγ may also lead to the collapse of the HF immune privilege and causes AA-like symptom (Fig. 3B). It was proposed that AA may represent multiple types of diseases rather than a single disease entity (Paus 2020).

The genetic basis of AA has been investigated in recent years through GWAS analyses (Petukhova et al. 2010, 2020; Betz et al. 2015). The most relevant genes include human leukocyte antigen (*HLA-DR*) or MHC class II, *IL2RA*, *IL2*/*IL21*, *CTLA4*, *Eos*, and *ULBP3*/*ULBP6* which is related to NKG2D-mediated T cell activity, and hair follicle antigens *STX17, PRDX5* and *BCL2L11*(*BIM*) (Fig. 3C). These data are consistent with the fact that AA is immune-mediated and is rooted on the collapse of the HF immune privilege. It was also proposed that the pigmentary system of the HF is particularly sensitive to immune attack, because the regenerated HFs in AA often produce white hair (Pratt et al. 2017). However, no loci related to melanogenesis were identified by GWAS at this moment.

Currently, AA is mainly treated by local or systemic corticosteroids, and local immune modulators which induce local inflammation or allergy (Fig. 3D; Pratt et al. 2017). Novel developments include restoring the HF immune privilege through local manipulation of the microenvironment, such as neutralizing IFNγ and providing αMSH/TGFβ1/IL10 (Paus 2020; Bertolini et al. 2020), or JAK inhibitors including ruxolitinib, tofacitinib and baricitinib to attenuate the immune attack (Pratt et al. 2017; Gilhar et al. 2019). However, relapse is common because the nature of the autoimmune attack is difficult to target by current methodology.

## Conclusions

Research on the regulation of HFSCs has seen rapid progress in recent years. We have gained knowledge regarding how the HFSCs respond to trauma, dietary factors, cold stress, hormone etc. We also start to appreciate the collective behavior of HFSCs, that is, hair regeneration is regulated at the population level rather than on each individual HF. The complex crosstalk among the different cell types within the skin during hair regeneration is of particular interest. Together, it appears the HFSCs represent an attractive model to quest the principles governing organ regeneration and the functional involvement of stem cells in general.

However, translational efforts to cure hair disorders have not been equally successful. The nowadays widely used hair loss remedies such as Minoxidil and Finasteride were developed long time ago when we knew very little about the molecular and cellular mechanism of hair growth and regeneration. Yet after decades of mechanistic study, we haven’t developed novel treatment options that are equally successful. This is partly due to the intrinsic complexity: both AGA and AA are of polygenetic origin. We even don’t have appropriate animal models to study these traits – although for AA there is a natural mouse model that can be used (Pratt et al. 2017), the genetic base of which is unclear and the relevance for human AA is uncertain. Human specimens and clinical trials remain the only convincing test for any appreciable progress. This appears to be a major hurdle for research and development. The fact that the most common forms of hair disorders, AGA and AA, are complex traits makes the study on their pathobiology and development of new treatment options much more interesting and challenging.

## Data Availability

Not applicable.

## Abbreviations

AA: Alopecia areata
AGA: Androgenetic alopecia
AR: Androgen receptor
DP: Dermal papilla
GWAS: Genome-wide association study
HF: Hair follicle
HFSC: Hair follicle stem cell
HG: Hair germ
